# Dosimetric feasibility of 4DCT-ventilation imaging guided proton therapy for locally advanced non-small-cell lung cancer

**DOI:** 10.1186/s13014-018-1018-x

**Published:** 2018-04-25

**Authors:** Qijie Huang, Salma K. Jabbour, Zhiyan Xiao, Ning Yue, Xiao Wang, Hongbin Cao, Yu Kuang, Yin Zhang, Ke Nie

**Affiliations:** 1 Department of Radiation Oncology, Rutgers-Cancer Institute of New Jersey, Robert Wood Johnson Medical School, New Brunswick, NJ USA; 2 Department of Medical Physics, Memorial Sloan Kettering Cancer Center, New York, NY USA; 3 Proton Therapy Center, University of Cincinnati Medical Center, Cincinnati, OH 45044 USA; 4 Department of Radiation Oncology, Shanghai Jiao Tong University School of Medicine Affiliated Renji Hospital, Shanghai, China; 5 Department of Medical Physics, University of Nevada, Las Vegas, NV USA

**Keywords:** 4D-CT ventilation imaging, Functional imaging guided proton therapy

## Abstract

**Background:**

The principle aim of this study is to incorporate 4DCT ventilation imaging into functional treatment planning that preserves high-functioning lung with both double scattering and scanning beam techniques in proton therapy.

**Methods:**

Eight patients with locally advanced non-small-cell lung cancer were included in this study. Deformable image registration was performed for each patient on their planning 4DCTs and the resultant displacement vector field with Jacobian analysis was used to identify the high-, medium- and low-functional lung regions. Five plans were designed for each patient: a regular photon IMRT vs. anatomic proton plans without consideration of functional ventilation information using double scattering proton therapy (DSPT) and intensity modulated proton therapy (IMPT) vs. functional proton plans with avoidance of high-functional lung using both DSPT and IMPT. Dosimetric parameters were compared in terms of tumor coverage, plan heterogeneity, and avoidance of normal tissues.

**Results:**

Our results showed that both DSPT and IMPT plans gave superior dose advantage to photon IMRTs in sparing low dose regions of the total lung in terms of V5 (volume receiving 5Gy). The functional DSPT only showed marginal benefit in sparing high-functioning lung in terms of V5 or V20 (volume receiving 20Gy) compared to anatomical plans. Yet, the functional planning in IMPT delivery, can further reduce the low dose in high-functioning lung without degrading the PTV dosimetric coverages, compared to anatomical proton planning. Although the doses to some critical organs might increase during functional planning, the necessary constraints were all met.

**Conclusions:**

Incorporating 4DCT ventilation imaging into functional proton therapy is feasible. The functional proton plans, in intensity modulated proton delivery, are effective to further preserve high-functioning lung regions without degrading the PTV coverage.

## Background

As confirmed by Radiation Therapy Oncology Group (RTOG) 9311 and several other clinical trials, dose-escalation is necessary and strongly associated with the improved survival for lung cancer patient receiving radiation treatment [1, 2]. However, tolerance of the normal lung to radiotherapy often limits the amount of radiotherapy that can be delivered to the primary cancer site. It showed that when concurrent chemotherapy was given in Stage III disease, more than 50% of patients developed Grade 3 or higher acute toxic effects and 10–15% of patients developed Grade 3 or higher chronic toxic effects [2]. Mean total lung dose, volume receiving 5 Gy (V5) and volume receiving 20 Gy (V20) are documented as being correlated with treatment-related toxicity. For example, radiation pneumonitis (RP), the most common complication of radiation therapy for non-small cell lung cancer (NSCLC), is found to be closely correlated with total lung V20. When V20 was over 22%, 32% and 40%, the incidence of radiation pneumonitis within 2 years increased from 0% to 7%, 13% and 36% respectively [3].

However, the current radiation planning practice assumes a uniform distribution of pulmonary function and homogeneous response to radiation. In fact, pulmonary function heterogeneity is present in lung cancer patients such as the lower lung has higher ventilation function levels than the upper lung [4]. Yorke et al. also demonstrated that the mean dose to the lower lung was more predictive of toxicity than that to the upper parts [5]. These findings suggested a radiation treatment strategy for avoidance of high-functioning lung, which might have the potential to reduce pulmonary toxicity thus to give possibility for dose-escalation. Previous studies have demonstrated decreasing the radiation dose to high-functioning lung areas and directing the radiation beams to the parts with perfusion/ventilation defects may help to protect highly functioning lung regions and thus reduce the incidence and seriousness of radiation pneumonitis (RP) [6–12]. There are even several on-going clinical trials as NCT02528942, NCT02308709, and NCT02843568 to evaluate the clinical outcome that utilizing functional imaging guided photon radiation to avoid more functional portions of the lung.

The presence of functional defects and advances in imaging techniques have led to an interest in utilizing functional imaging modalities such as single photon emission computed tomography (SPECT) [13, 14], hyperpolarized Helium or Xenon MRI [15, 16], and 4-dimensional (4D) computed tomography (CT) ventilation imaging [7, 9, 17], to identify high functioning lung for the purpose of preferential radiation sparing. Because 4D-CT imaging is increasingly a standard for lung cancer treatment, it can be implemented with little additional radiation or cost to the patients. Previous studies have detailed the methodology of 4D-CT ventilation imaging [7, 17], the validation of the technique [18–21], and its potential clinical uses as a functional imaging modality [8, 10, 22, 23].

However, to our best knowledge, all previous studies focused on the feasibility of functional imaging guided photon treatment either using 3D-conformal radiation therapy (CRT) or IMRT techniques. It is known that, proton therapy has the physical properties to deliver minimal dose beyond distal end of the Bragg peak, which generally allows for reduced doses to organs at risk compared to photon therapy. In this study, we take functional imaging guided therapy one step further to investigate the feasibility of incorporating pulmonary function information derived from 4D-CT data into proton planning, and to compare the benefits of sparing high-functioning lung in both functional-guided double scatter proton therapy (DSPT) and intensity modulated proton therapy (IMPT) for locally advanced non-small-cell lung cancer (NSCLC).

## Methods

### Patient selection and CT imaging data

This study was approved by the Institutional Review Board. Ten consecutive patients (6 female) with locally advanced non-small cell lung cancer (NSCLC) who underwent proton treatment at our institutes were selected. Each patient had clinically-approved double scatter proton treatment (DSPT) plan and an IMRT plan designed for insurance purpose. Patient’s plan was revisited and redesigned later with intensity modulated proton treatment (IMPT) capability. In our clinic, if the patient breathing motion is less than 10 mm measured from CT0 (end-of-inspiration) to CT50 (end-of-expiration), then the patient can be recommended for DSPT treatment. While the clinic criterion in screening patients suitable for IMPT is tighter compared to DSPT treatment, patients breathing motion should be limited to 5–7 mm from CT0 (inhalation) to CT50 (max-exhalation) as suggested by Kardar et al. and Li et al. [24, 25]. Only 8 of 10 patients met this criterion and thus used for analysis. Table 1 lists the patient characteristics, the tumor location, tumor size, and prescribed radiation dose (Rx).

**Table 1:** Patient Characteristics

	Age (y/o)	Gender	Stage	Location	Motion (mm)	CTV Volume (cm^3^)	Rx (cGy/fx x fxs)
#1	87	F	IIIa	LML	4.6	349.4	200 × 33
#2	59	F	IV	LUL	1.4	265.1	200 × 33
#3	48	M	IIIa	LML	4.6	429.3	200 × 30
#4	65	M	IIIb	RUL	1.3	562.4	200 × 27
#5	67	F	III	RML	4.8	738.4	200 × 33
#6	79	F	IIIa	RML/RLL	5.2	265.9	200 × 30
#7	84	M	IV	LML	3.1	572.2	200 × 30
#8	61	F	IIIa	RML	2.7	435.6	200 × 30

*y/o* years old, *RUL* right upper lobe, *RML* right middle lobe, *RLL*, right lower lobe, *LUL*, left upper lobe, *LML* left middle lobe, *LLL* left lower lobe

All patients had undergone 4D-CT scanning for treatment planning purpose. The 4D-CT data sets were obtained using a 16-detector row spiral CT scanner (Lightspeed 16; GE Medical Systems, Milwaukee, WI, USA) in cine mode with a 2.5-mm slice covering the entire chest. All received vocal coaching with the Varian Real-time Position Management system (Varian Medical Systems, Palo Alto, CA) to promote regular respiration during 4D-CT acquisition. Image bins corresponding to 10 phases of the respiratory cycle were created. A medical physicist was present during each CT scan to ensure that the patient’s breathing was not erratic.

### 4D-CT based ventilation imaging

The detail methodology of deriving ventilation map from 4D-CT was outlined by Yamamoto et al. [8]. In brief, paired 4D-CT images at the peak-exhale and peak-inhale phases were used for ventilation computation. The displacement vector field (DVF) was firstly generated using deformable image registration (DIR). Then the Jacobian determinant of DVF was created as a ventilation metric. The regional ventilation, V (x, y, z), is given by:

1 $$ V\left(x,y,z\right)=\left|\begin{array}{ccc}\frac{1+\partial {u}_x\left(x,y,z\right)}{\partial x}& \frac{\partial {u}_x\left(x,y,z\right)}{\partial y}& \frac{\partial {u}_x\left(x,y,z\right)}{\partial z}\\ {}\frac{\partial {u}_y\left(x,y,z\right)}{\partial x}& \frac{1+\partial {u}_y\left(x,y,z\right)}{\partial y}& \frac{\partial {u}_y\left(x,y,z\right)}{\partial z}\\ {}\frac{\partial {u}_z\left(x,y,z\right)}{\partial x}& \frac{\partial {u}_z\left(x,y,z\right)}{\partial y}& \frac{1+\partial {u}_z\left(x,y,z\right)}{\partial z}\end{array}\right|-1 $$

**Fig. 1:**
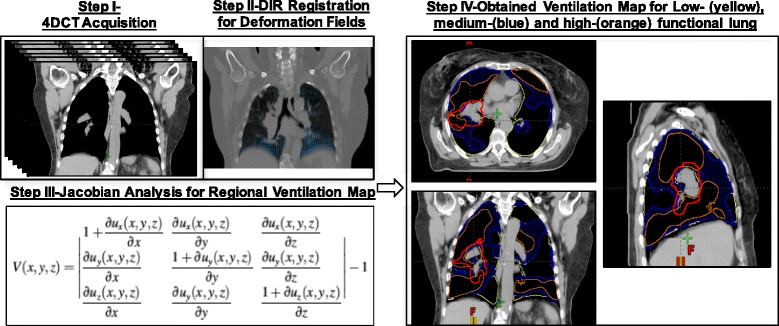
Schematic diagram for creating anatomical treatment plan and 4DCT ventilation image-based functional plan and quantifying impact of functional planning. Step I-4DCT image acquisition; Step-II: to obtain the deformation vector fields (DVFs) using deformable image registration on 4DCT images; Step-III: Jacobian analysis of the DVFs to obtain regional ventilation map; Step-IV: segmentation of three functional lung regions with equal volumes: low-functional (yellow), medium-functional (blue), and high-functional (orange) lung with ITV showing red. The obtained result is used to create functional proton plans to further avoid high-functional lung regions

Where u(x,y,z) is the DVF of the voxel at location (x,y,z) of a peak-exhale image to the corresponding location of a peak-inhale image. Regional ventilation was determined for each voxel at the peak-exhale phase, resulting in a 4D-CT ventilation map. Then the ventilation values outside the lung regions were zeroed out. Two threshold values were identified for each case to further divide the total lung into three equal volumes, as low-, medium-, and high-functional lungs throughout our report. An example of the functional lung of ventilation mapping procedure was shown in Fig. 1.

As the 4D-CT derived ventilation map could vary with the DIR algorithms, the validity of this method has been published in one of our previous works [26–28]. Briefly, the DIR generated ventilation map was directly compared with that captured by hyperpolarized gas tagging MRI on real patients. A large number (300–500) of uniformly distributed landmarks were identified throughout the entire lung. A ground truth of DVF was obtained by tracking the location of each tagged grid between the exhalation and the inhalation images. The DVF generated from seven DIR algorithms were compared to the ground truth DVF included Velocity, MIM, Mirada, Elastix and 3 other in-house built algorithms from DIRART toolbox as Double Force Demons, Improved Lucas-Kanade, and Iterative Optical Flow. Among all algorithm, the Multi-pass B-spline based deformable image registration from Velocity™ (Varian, Palo Alto, CA) gave the most reasonable result, which was thus used in this study.

### Anatomical and functional treatment planning

Two treatment-planning scenarios were included in this study: anatomical plans based on the constraints on the total lung and functional plans using ventilation image-guided information to avoid high-functioning lung. A total of 5 plans were created for each individual patient to quantify the impact of functional proton planning: (1) anatomical IMRT (_a_IMRT), (2) anatomical double scattering proton treatment (_a_DSPT), (3) functional double scattering proton treatment (_f_DSPT), (4) anatomical intensity modulated proton treatment (_a_IMPT), and (5) functional intensity modulated proton treatment (_f_IMPT). Contouring and treatment planning were performed using the Varian Eclipse treatment planning system.

The target delineation and dosimetric constraints were followed with RTOG 1308. The gross tumor volume (GTV) was defined on the average CT image. The clinical target volume (CTV) was defined as GTV plus an 8-mm margin for micro extension of the tumor. An internal target volume (ITV) was the union of the CTV plus motions from all phases of respiration and edited clinically based on pattern of tumor spreading and anatomic boundaries such as vertebral body, chest wall and heart etc. The planning target volume (PTV), defined as an expansion of the ITV by 5 mm, was used for IMRT planning and all plans evaluation. Critical organs, including the heart, spinal cord, esophagus, and total lungs, were contoured on the average CT image. The spinal cord and esophagus were expanded by 5 mm for planning purpose. The planned dose as calculated on the average CT was defined as the 3D dose. All the plans were designed to deliver 60–70 Gy (RBE: relative biological effective dose) with 2 Gy per fraction. The dose constraints were given in Table 2.

**Table 2:** Treatment planning goals and constraints

Structure	Constraints
PTV	No less than 95% coverage by prescription dose
Total Normal Lung	V20 ≤ 37%
	V5 ≤ 60%
	MLD (Mean Lung Dose) ≤ 20Gy
Spinal Cord	Dmax < 50 Gy
Heart	Dmean< 26 Gy
Esophagus	Dmean <34Gy

For photon IMRT planning, the PTV was treated with any combinations of coplanar or non-coplanar fields shaped and modulated to deliver the specified dose while restricting the dose to normal tissues. Beam weighting and modulation were determined by inverse IMRT planning procedures. Photon plans were optimized to cover the PTV. For proton DSPT treatment planning, a plan with 2 or 3 fields was generated. The proton was modeled to have a maximum energy of 250 MeV. The relative biologic effectiveness is taken to be 1.1. To compensate for the perturbation of the proton dose distribution due to the conversion uncertainty of CT numbers to proton stopping power, and the beam range uncertainty for energy, variable scattering system thickness, and compensator density etc., the compensator was smeared using the algorithms established by Moyers et al. [29]. If ITV was used as the planning target, the distal and proximal margins in beam direction were determined by distal (proximal) *m* = 0.035 *R* + 3 mm, where *R* was the most distal (proximal) water equivalent depth of the target volume. If the PTV coverage was less than 95% coverage under the ITV-based planning, then the PTV itself was used as the planning target to ensure better coverage. In this situation, smearing margins were considered to be zero [29]. To reduce hotspot, a 1-cm border-smoothing margin was further applied to the compensator. After the proton beams were designed, dose was calculated on the average CT. For IMPT treatment planning, the dose engine used proton pencil beams nominally defined in the 70 to 227 MeV energy range. The spot size ranged from 3.2 mm (1 sigma) at the isocenter in air at the maximum energy level to 7.5 mm at the lowest energy level. The spot spacing was fixed with 5 mm and ITV was used as the planning target. Robust optimization with worst-case scenario, as first introduce by Lomax et al. [30], was applied for each individual case. For any given beam arrangement, nine different dose scenarios were calculated simultaneously: the nominal dose distribution (i.e., that with no consideration of uncertainties) and dose distributions incorporating (1) set-up uncertainties, obtained by shifting the isocenter of the CT images by 5 mm along the anterior-posterior, left-right, and superior-inferior directions (yielding 6 dose distributions); and (2) range uncertainty, by scaling the relative stopping power ratios to water by 3.5% (yielding additional 2 dose distributions). The worst-case dose distribution was then represented by the minimum of the 9 doses in each voxel in the ITV and the maximum of the 9 doses in each voxel outside the ITV. A difference of 5% between the worst-case dose distribution and the nominal dose was considered as acceptable. If the plan was found to be not robust (quantified by a > 5% difference), then the plans were re-optimized. The treatment plan was ultimately deemed acceptable if 95% of the PTV was covered by the prescription dose.

For all the cases, the anatomically constrained plans were generated blind of pulmonary ventilation information, basically treating the lung as a whole. The functionally constrained plans were generated with additional constraints to further reduce the dose to the high functioning lung regions. Functional DSPT was achieved by changing the gantry angles to avoid critical OARs and highly functioning lung regions or by smoothing the compensators. Functional IMPT was generated with additional constraints to reduce the V5 and V20 of the identified functional regions. In order to allow for a direct comparison, the number of beams and beam angels were kept identical for _a_DSPT vs. _a_IMPT plans. All plans were normalized so that the D95 of the PTV equaled the prescription dose. Also the hotspot inside the PTV was limited to 115% of the prescription dose.

### Statistical analysis

Statistical analyses were performed to quantify the impact of proton treatment vs. photon treatment and functional planning vs. anatomical planning. The percentage of lung volume receiving > 5 Gy, and > 20 Gy were calculated for the functional lung and total lung. Regarding the other critical structures, the maximum dose (Dmax) delivered to the spinal cord, the mean dose (Dmean) to esophagus, the mean dose to the heart were also quantified.

## Results

### Comparison of all treatment plans for an example patient

**Fig. 2:**
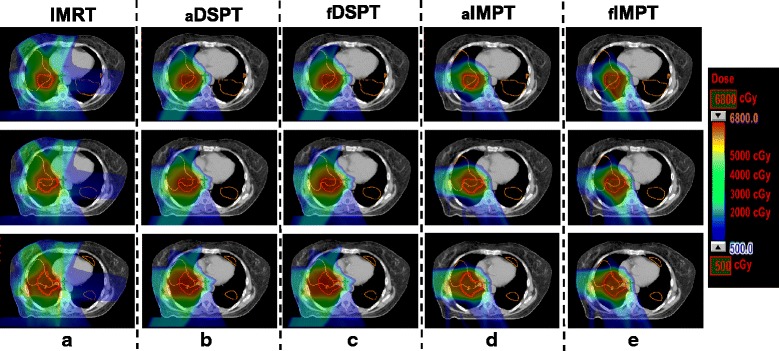
Isodose distributions of an example patient showing multiple slices, from (**a**) IMRT, (**b**) anatomical double scattering proton treatment (_a_DSPT), (**c**) functional double scattering proton treatment (_f_DSPT), (**d**) anatomical intensity modulated proton treatment (_a_IMPT), (**c**) functional intensity modulated proton treatment (_f_IMPT) plans. The PTV is highlighted in red and high-functional lung regions are in orange

Figure 2 shows the isodose distribution of an example patient. As there was minimal exit dose for proton treatment, all proton plans showed clear sparing of the contralateral lung compared to the IMRT plans. In addition, functional IMPT planning spared the high-functioning lung further compared with anatomical planning. In particular, the intermediate dose areas (2000 cGy – 5000 cGy) were distorted toward to areas with less high-functioning lung. Functional DSPT planning showed considerable distortions of the isodose curves around the PTV compared with anatomical DSPT but the difference was not significant. The total lung volumes receiving over 20 Gy (V20) was comparable between IMRT (38.4%) and DSPT (35.5% for _a_DSPT, 35.4% for _f_DSPT) but reduced to 24.3% (25.2%) with _a_IMPT (_f_IMPT) treatment for this example case. The total lung receiving 5Gy (V5) were significantly reduced from 78.9% for IMRT to 44.9% of _a_DSPT and 40.1% with _a_IMPT. The high-functioning lung receiving 5Gy was reduced from 80.2% (IMRT) to 47.6% with _a_IMPT, and further to 43.3% with functional IMPT. The high-functioning lung V20 was 41.6% from IMRT, reduced to 37.7% for _a_IMPT and further to 34.6% for _f_IMPT. In addition, functional planning did not result in increases in heart dose. The mean dose of esophagus increased slightly from 9.6 Gy to 10.7 Gy from _a_IMPT to _f_IMPT, but remained unchanged as 0.8 Gy for both anatomical and functional DSPT planning. The cord dose was 37.1 Gy for IMRT, reduced with proton treatment to 15.8 Gy for _a_DSPT and remained unchanged with _f_DSPT. Although with functional IMPT planning, the cord max dose increased from 28.1 Gy (_a_IMPT) to 31.5Gy (_f_IMPT), it still met the dose constraint (Dmax < 50Gy).

### Comparison of all treatment plans for all patients

**Fig. 3:**
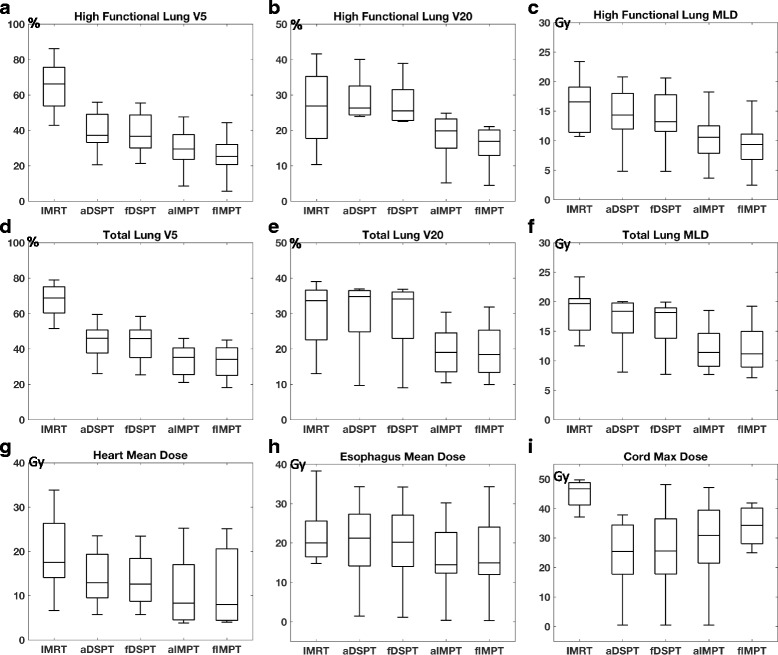
The box plot of dosimetric metrics across IMRT, anatomical DSPT, functional DSPT, anatomical IMPT and functional IMPT, (**a**) high functioning lung V5, (**b**) high functioning lung V20, (**c**) high functioning lung mean lung dose (MLD), (**d**) total lung V5, (**e**) total lung V20, (**f**) total lung mean lung dose (MLD), (**g**) heart mean dose, (**h**) esophagus mean dose, (**i**) cord max dose, respectively

Only 2/8 IMRT plans were clinically acceptable. Since most of the tumors were located mediastinum or middle lobe, achieving acceptable low V5 was the limiting factor for most of the IMRT plans. The dosimetric metrics for doses to critical structures are shown in Fig. 3. Both DSPT and IMPT plans showed superior dose advantages to photon IMRTs in sparing low dose regions of the total lung. The functional planning in IMPT delivery, can further reduce the low dose in high-functioning lung without degrading the PTV dosimetric coverages, compared to anatomical proton planning. The median reductions in the percentage of volume irradiated with > 5 Gy, and > 20 Gy in high-functioning lung were 32.5% [15.4%–77.6%] and 5.2% [− 3.9% -15.6%] for anatomical IMPT plans, and further to 36.8% [21.0%–80.5%] and 7.2% [− 1.5%–17.6%] for functional IMPT plans compared to IMRT. Although the doses to several other critical organs might increase during functional planning, the necessary constraints were all met. For double scattering technique, the V20 of the total lung is not necessarily lower but V5 was much lower with median of 21.6% [6.6%–42.6%] reduction when compared to IMRT. The functional DSPT only showed marginal decrease in both high-functioning lung V5 and high-functioning lung V20 compared to anatomical plans.

## Discussion

In this study, we evaluated the impact of accounting spatial heterogeneity in lung function using 4D-CT based ventilation imaging for guided proton treatment to further avoid high-functioning lung. Our results demonstrate that functional proton planning, especially in intensity modulated spot-scanning delivery technique, can further reduce the low-dose in high-functional lung regions compared to anatomical proton planning. Although the doses to several other critical organs might increase during functional planning, the necessary constraints were all met. The study presented here demonstrated the potentials of proton treatment in lowering the dose to the normal lung and, for the first time demonstrated the benefit of using 4D-CT ventilation imaging to guide the proton treatment to further sparing high-functional regions.

Current RT planning aimed at limiting lung toxicity assumes a uniform response to radiation and fails to take account of spatial and temporal pattern. Actually, different sections of lung could have different levels of pulmonary functions [4]. Decreasing the radiation dose to functional lung areas and directing the rays to the parts with perfusion/ventilation defects may help to protect highly functional regions and thus reduce the incidence and seriousness of radiation pneumonitis [6–12]. Functional imaging-guided radiotherapy with the purpose of local boost or functional avoidance of normal organs is increasingly used in clinics. Lavrenkov et al. compared functional IMRT planning and functional 3D-CRT planning using single photon emission CT (SPECT) perfusion images guided treatment and demonstrated that functional IMRT planning led to a lower high functioning lung mean dose than functional 3D-CRT planning [31]. Yamamoto et al. demonstrated that 4D-CT ventilation imaging based functional IMRT and VMAT treatment planning, by changing only one variable (i.e., absence/presence plan), led to significant reductions in the high-functioning lung dose [8]. However, previous work was mainly done with regular photon treatment. The energy distribution of protons as opposed to photon-based irradiation has theoretical advantages because of the Bragg peak of proton particles, which can be exploited to reduce exposure of normal tissues to radiation, particularly at low doses. Under this premise, emerging dosimetric and clinical studies are being undertaken to assess the role of proton radiotherapy vs. photon and the associated clinical outcome [32–34]. Several studies have reported how NSCLC proton therapy reduces the dose to the normal lung, heart, esophagus, and spinal cord compared with IMRT, while delivering an escalated dose [35]. Furthermore, a retrospective study demonstrated that survival rates and high local control rates can be potentially improved without enhancing radiation-induced toxicities using proton therapy and concurrent chemotherapy [36]. To our best knowledge, it is the first time that functional ventilation imaging derived from 4D-CT was utilized to guide proton treatment. We found functional proton treatment, in IMPT delivery, reduced the dose for high functioning lung without significantly increasing the doses to the other critical organs compared to anatomic planning or at the expense of significantly degrading the target coverage. While the functional DSPT only showed marginal benefit in sparing low dose to the high-functioning lung compared to anatomical plans. But the proton treatment was in general giving less low-dose to the total lung in terms of V5 compared to IMRT treatment. Overall, these results might promote future dose-escalation studies with functional imaging guided proton therapy for lung cancer treatment.

Before applying to future clinical studies, two major aspects need to be addressed. First is the validation of 4D-CT derived ventilation imaging in depict patient pulmonary function. There have been several studies attempted to validate 4DCT-ventilation by comparing it against other ventilation imaging modalities such as nuclear medicine ventilation-perfusion imaging [19], xenon-CT [14, 37], PET [38], and MRI [39] or directly with pulmonary function test [20]. The studies generally found good agreement on a global level. Yet the regional physiologic accuracy has not been validated in patients. In addition, temporal changes in regional ventilation to a segment of lung previously impaired by compression from a local tumor might occur during the course of radiation treatment. A possible explanation of these changes is that the shrinkage of lung tumor in response to radiation might increase the ventilation due to reopening of the airways [40]. Nevertheless, additional work is needed to validate the regional physiologic accuracy of 4DCT derived ventilation imaging in real patients especially during the course of radiation treatment.

Secondly, proton radiotherapy in lung cancer raises several issues. Among those, the most challenge one is the tumor motion during treatment from patient’s breathing. To take that into consideration, the target volume was designed on 4D-CT based ITV with consideration of all respiratory motion. To further reduce the chance of missing the target even when the target moves, a relative large smearing margin as recommended by Moyers et al. [29] for highly mobile lung tumors was utilized in double scattering proton therapy (DSPT). This approach might slightly over treat the normal tissues behind the tumor when tumor moves out of the field. However, it would make sure that whole tumor be treated adequately no matter where it is located during the different breathing phases. On top of that, robust optimization with worst case scenario was utilized in all proton planning to evaluate the setup uncertainties and range uncertainties. Regarding plan evaluation, using the 4D composite doses calculated with 4D-CT images and deformable image registration (DIR) is a well-accepted method of evaluating the actual delivered dose for DSPT in the presence of respiratory motion [41]. It is our clinical practice to firstly evaluate the patient breathing motion based on the fluorescence and 4D scans. If the breathing motion is less than 10 mm measured from CT0 (end-of-inspiration) to CT50 (end-of-expiration), then the patient can be recommended for DSPT treatment. And then the plan generated on the average CT is transferred to the CT0 and CT50 using deformable image registration. Only if the target coverage differences between these two extreme phases is less than 5%, the plan is considered as robust. For the IMPT technique, the most concern comes from the interplay effects between the moving beams and moving tissue. The magnitude of the interplay effect with scanning proton beams has been reported in previous studies, and it has been shown that proton dose could be impacted enormously by the interplay effect for tumor motions around or larger than 10 mm [42–47]. Kardar et al. and Li et al. introduced a 4D dynamic dose simulator and further investigated the impact of motion pattern and starting phases on the interplay effects [24, 25]. They observed situations in which motion more than 5 mm and small tumor sizes led to relatively large uncertainties caused by the interplay effect in a single fraction. In contrast, for some patients with motion less than 5 mm and large tumor size treated with multi-fractionation, the interplay effect was small. Moreover, a recent study by Inoue et al. evaluated the impact of setup and range uncertainties, breathing motion, and interplay effects in IMPT dose distributions [48]. Their results demonstrated that in robust-optimized plans the dosimetric effects due to geometric and radiologic variation had a limited impact on target coverage, target dose homogeneity, and organ-at-risk dose parameters if treated with multi-fractionation. As such, in our clinical practice, we selected patients with breathing motion less than 5–7 mm but not small sizes of lung tumors for IMPT. In addition, we compared the target coverage difference between the CT0 and CT50 using a threshold of 5% as suggested by Kardar et al. and Li et al. [24, 25]. Only those patients (8/10 in this study) met the selection criterion were considered for IMPT treatment. Overall, further studies are needed to guarantee the robust delivery of the proton treatment in 4D scenarios.

Nevertheless, as shown in our study, proton treatment still reduced the doses to the normal tissues, especially the low-dose exposure compared with IMRT. This may have significant impact in reducing lung toxicity. Currently, there is insufficient outcome data to confirm the correlation between functional lung dose-volume parameters and pulmonary toxicity endpoints, and further studies are needed to determine if dosimetric reductions to functional lung will translate into clinical benefits for NSCLC patients. In addition, in a very recent review paper by Ireland et al., that there may be patients with specific types of functional defects and tumor volumes and positions will benefit from the inclusion of functional data for normal lung dose reduction [49]. It will be very interesting in the future to recruit more patients to further identify the sub-cohort of the patients who will benefit from not just proton treatment but also functional data guided proton treatment.

## Conclusions

In conclusion, this study demonstrated that incorporating 4DCT-based pulmonary ventilation information into functional proton therapy is feasible. The functional proton plans, in IMPT delivery, were effective to furtherly preserve high-functioning lung regions without degrading the PTV coverage. Although the doses to some critical organs might increase during functional planning, the necessary constraints were all met.

## Abbreviations

4D-CT: 4-dimensional (4D) computed tomography
_a_DSPT: Anatomical double scattering proton treatment
_a_IMPT: Anatomical intensity modulated proton treatment
_a_IMRT: Anatomical IMRT
CRT: Conformal radiation therapy
CTV: Clinical target volume
D95: 95% of the volume received dose
DIR: Deformable image registration
Dmax: Maximum dose
Dmean: Mean dose
DVF: Displacement vector field
_f_DSPT: Functional double scattering proton treatment
_f_IMPT: Functional intensity modulated proton treatment
GTV: Gross tumor volume
ITV: Internal target volume
NSCLC: Non-small cell lung cancer
PTV: Planning target volume
RBE: Relative biological effective
RP: Radiation pneumonitis
RTOG: Radiation Therapy Oncology Group
SPECT: Single photon emission computed tomography
V20: Volume receiving 20 Gy
V5: Volume receiving 5 Gy
